# Loggerhead Sea Turtle as Possible Source of Transmission for Zoonotic Listeriosis in the Marine Environment

**DOI:** 10.3390/vetsci10050344

**Published:** 2023-05-11

**Authors:** Silva Rubini, Matilde Baruffaldi, Roberta Taddei, Giulia D’Annunzio, Erika Scaltriti, Martina Tambassi, Ilaria Menozzi, Giulia Bondesan, Sandro Mazzariol, Cinzia Centelleghe, Giorgia Corazzola, Federica Savini, Valentina Indio, Andrea Serraino, Federica Giacometti

**Affiliations:** 1 Laboratory of Ichthyopathology and Marine Biotoxins, Zooprophylactic Institute of Lombardia and Emilia Romagna Regions (IZSLER), 44124 Ferrara, Italy; silva.rubini@izsler.it (S.R.); matibaruffaldi@gmail.com (M.B.); roberta.taddei@izsler.it (R.T.); giulia.dannunzio@izsler.it (G.D.); 2 Risk Analysis and Genomic Epidemiology Unit, Zooprophylactic Institute of Lombardia and Emilia Romagna Regions (IZSLER), 43126 Parma, Italy; erika.scaltriti@izsler.it (E.S.); martina.tambassi@izsler.it (M.T.); ilaria.menozzi@izsler.it (I.M.); 3 Independent Contractor Veterinary and Delta Rescue President, 44020 Ferrara, Italy; giuliabondesan@libero.it; 4 Department of Comparative Biomedicine and Food Science—BCA, University of Padua—Agripolis, 35020 Padua, Italy; sandro.mazzariol@unipd.it (S.M.); cinzia.centelleghe@unipd.it (C.C.); giorgia.corazzola@unipd.it (G.C.); 5 Department of Veterinary Medical Sciences, University of Bologna, 40064 Bologna, Italy; valentina.indio2@unibo.it (V.I.); andrea.serraino@unibo.it (A.S.); federica.giacometti3@unibo.it (F.G.)

**Keywords:** *Listeria monocytogenes*, sea turtle, heterophilic granuloma, whole genome sequencing, sequence type 6

## Abstract

**Simple Summary:**

*Listeria monocytogenes* is a widespread bacterium in nature thanks to its broad pH range stability as well as toleration of salt conditions up to 20% and temperatures ranging from −0.4 to 45 °C. The bacterium causes infection both in animals and humans, with variable and sometimes fatal disease manifestations. We isolated *L. monocytogenes* from a stranded loggerhead sea turtle (*Caretta caretta*) that perished briefly after being rescued due to the lesions in many internal organs. Our findings underline how the sea turtle and its marine environment should be considered a source of *L. monocytogenes* contamination. This is important, given zoonotic transmission either through contact with animals or via contaminated food, as well as in the light of the migratory behavior of *Caretta caretta*.

**Abstract:**

*Listeria monocytogenes* is an ubiquitous pathogen isolated from different host species including fish, crustaceans, and molluscs, but it is rarely a pathogenic microorganism to marine reptiles. In particular, only two cases of fatal disseminated listeriosis have been described in the loggerhead sea turtle (*Caretta caretta*). In this study, we describe a lethal case of *L. monocytogenes* infection in a loggerhead sea turtle. The turtle was found alive, stranded on a beach in North-eastern Italy, but perished soon after being rescued. The autoptic examination revealed that heart, lung, liver, spleen, and urinary bladder were disseminated with multiple, firm, 0.1–0.5 mm sized, nodular, white-green lesions. Microscopically, these lesions corresponded with heterophilic granulomas with Gram+ bacteria within the necrotic center. Furthermore, the Ziehl–Neelsen stain was negative for acid-fast organisms. Colonies isolated from heart and liver were tested through MALDI-TOF for species identification, revealing the presence of *L. monocytogenes*. Whole Genome Sequencing on *L. monocytogenes* isolates was performed and the subsequent in silico genotyping revealed the belonging to Sequence Type 6 (ST 6); the virulence profile was evaluated, showing the presence of pathogenicity islands commonly observed in ST 6. Our results further confirm that *L. monocytogenes* should be posed in differential diagnosis in case of nodular lesions of loggerhead sea turtles; thus, given the zoonotic potential of the microorganism, animals should be treated with particular caution. In addition, wildlife animals can play an active role as carriers of possibly pathogenetic and virulent strains and contribute to the distribution of *L. monocytogenes* in the environment.

## 1. Introduction

Due to its widespread distribution in nature, *Listeria monocytogenes* is found in diverse substrates such as plants, soil, silage, sewage, and water and in a wide range of animals. Indeed, the bacterium can persist in a variety of environments thanks to its broad pH range stability (between 4.6 and 9.5) as well as toleration of salt conditions up to 20% and a temperature range from −0.4 to 45 °C [1,2]. Thus *L. monocytogenes* and non-pathogenic *Listeria* spp. have been isolated from irrigation and natural waters with loads varying from 3 to 15 CFU ml^−1^ [3] to over 10^3^ CFU ml^−1^ [4].

*L. monocytogenes* is a food-borne zoonotic pathogen and can cause listeriosis in humans with variable, sometimes fatal (20–30%) disease manifestations, where most common invasive forms are neurolisteriosis, bacteremia, and pregnancy-related infection including neonatal listeriosis [5]. It can also infect a wide host range of domestic and/or wild mammals including birds, fish, reptiles, and crustaceans [6,7,8,9]. Most of the time, infection in animal hosts is subclinical (asymptomatic carriers) but severe forms can also occur [10] in the form of the three main manifestations listed above. In animals, listeriosis is primarily a disease of ruminants, and small ruminants, especially sheep, are mostly affected. Septicemic disease occasionally occurs in horses and pigs, up to sporadic/rare in poultry, while outbreaks of listeriosis are uncommon in birds. Few cases of reptilian listeriosis have been documented to date; specifically, only two other cases of fatal disseminated listeriosis [11,12] have been described at approximately the same time period in loggerhead sea turtles (*Caretta caretta*), both in Italy. Here we report an additional description in which a possibly pathogenic *L. monocytogenes* strain was isolated from different organs of an animal that presented nodular lesions within the whole celomatic cavity.

## 2. Materials, Methods, and Results

**Figure 1:**
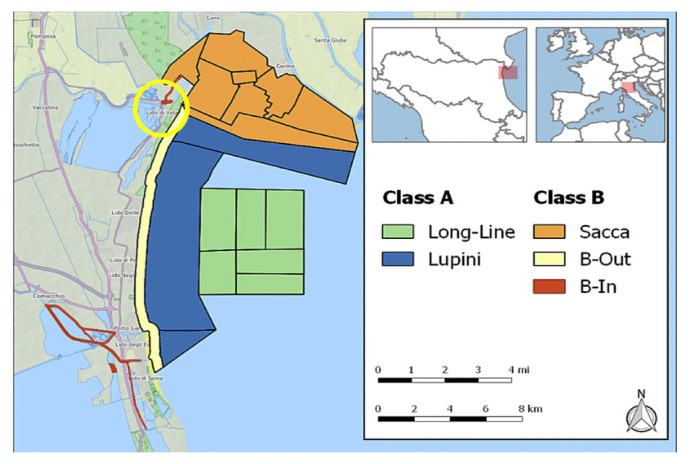
Map of area where the animal was found stranded (yellow circle).

In the morning of 20 May 2022 an adult female loggerhead sea turtle, 33 kg in weight with a curved carapace length (CCL) of 68.5 cm, was found stranded and alive in Lido di Volano beach (Ferrara, North-eastern Italy, 44°49′06″ N 12°17′08″ E, see also Figure 1). The animal was found on the beach while heading inland and immediately rescued. The discovery area is the shoreline, with stretches for free beach or equipped with bathing establishments in the north-west of the Adriatic Sea facing the Po river delta, the major Italian river which, from spring to estuary, flows through the Po Valley (Pianura Padana). First aid was given to the animal in the closest sea turtle first aid center of Goro, Ferrara.

**Figure 2:**
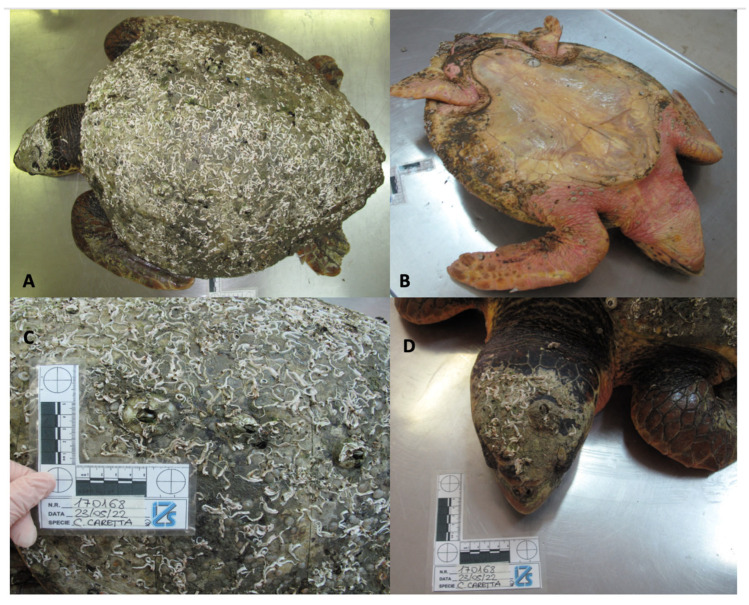
Dorsal (**A**) and ventral (**B**) views of the animal after death. Detail of the *Serpulidae* worms and *Balanomorpha* barnacles present on the carapace (**C**) and on the head (**D**) of the animal.

The turtle did not show external lesions but was hyporeactive to external stimuli. The clinical examination allowed observation of an accelerated respiration rate of one breath every 30 s (normal respiratory rate is 0.5 to 0.7 breaths/minute), and diffuse crepitus on auscultation of the cranial lung fields. The animal was treated with an intracoelomatic infusion of 1.5 mL/kg of physiological solution, 1.5 mL/kg of ringer solution, 1.5 mL/kg of glucose 5%, 10 mg/kg Baytril, and 1 mL/kg of Metabolase. Unfortunately, the death occurred the same day, eleven hours after being rescued. The carcass was immediately submitted to the Istituto Zooprofilattico Sperimentale della Lombardia e dell’Emilia Romagna for autoptic investigation. At gross examination, the carcass presented a body condition score of 2 (Figure 2A), based on the Net Cet [13] subjective body condition scoring system, thus meaning: “Moderate decomposition. Bloated carcass with characteristic mild odor. Head: integral or with partial loss of skin; eye: sunken or liquefied; tail: present or absent; limbs: integral; carapace and plastron: integral.”

The ventral portion of the neck showed erythematous and reddened skin (Figure 2B), and greenish discharge oozed from the oral cavity. During the external examination, the carapace appeared multifocally covered by numerous *Serpulidae* worms and barnacles (Order: *Balanomorpha*), the latter also attached to the soft skin (Figure 2C,D).

**Figure 3:**
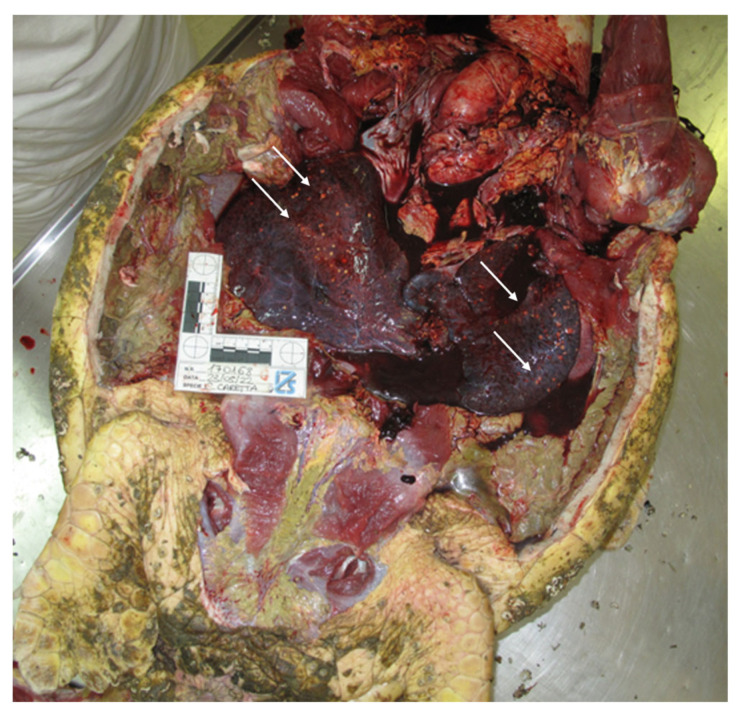
Presence of multifocal, white-green, nodular lesions, identified with arrows, affecting visceral organs.

**Figure 4:**
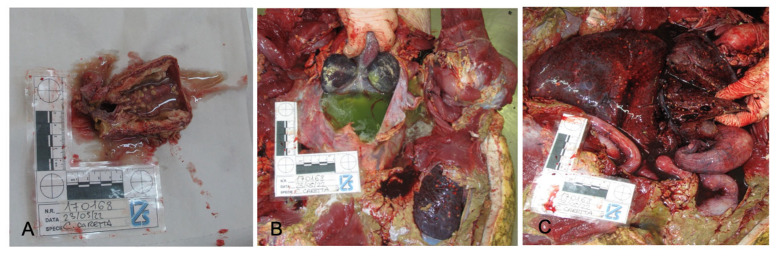
Organ details showing nodular lesions on urinary bladder (**A**) and liver (**C**), and pericardial effusion (**B**).

The examination of the internal organs revealed multiple, firm, 0.1–0.5 mm sized, nodular white-green lesions of heart, lung, liver, spleen, and urinary bladder and gelatinous pericardial effusion (Figure 3 and Figure 4A–C), as well as the presence of gastrointestinal helminths in the lumen of the esophagus and stomach. No other gross lesions were observed.

**Figure 5:**
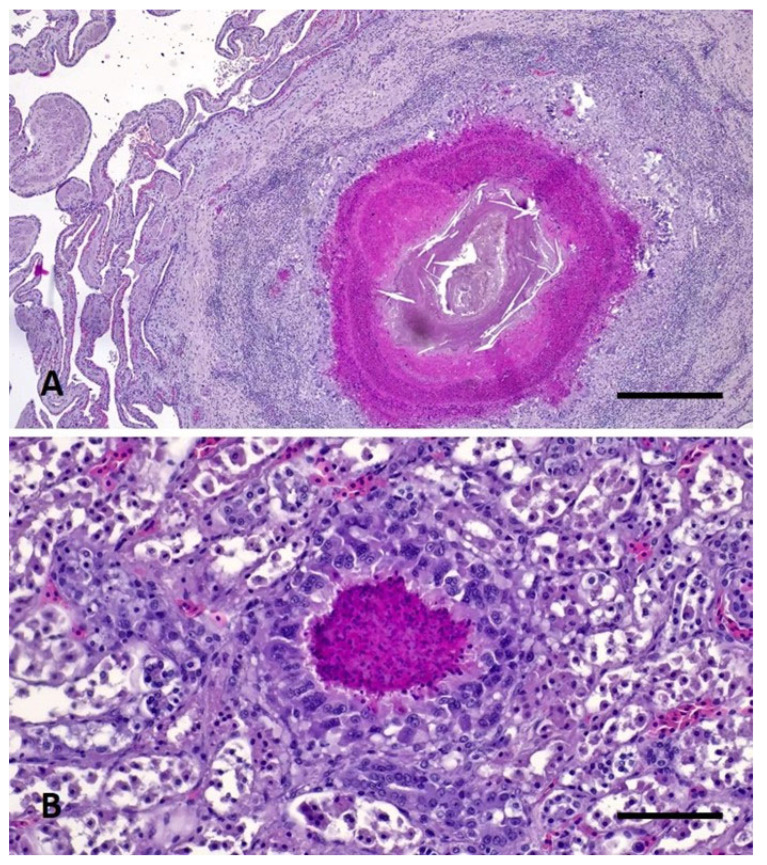
Heterophilic granulomas in pulmonary tissue (**A**) and kidney (**B**). Hematoxylin and eosin stain. Magnification 200×, scale bars: 1 mm.

**Figure 6:**
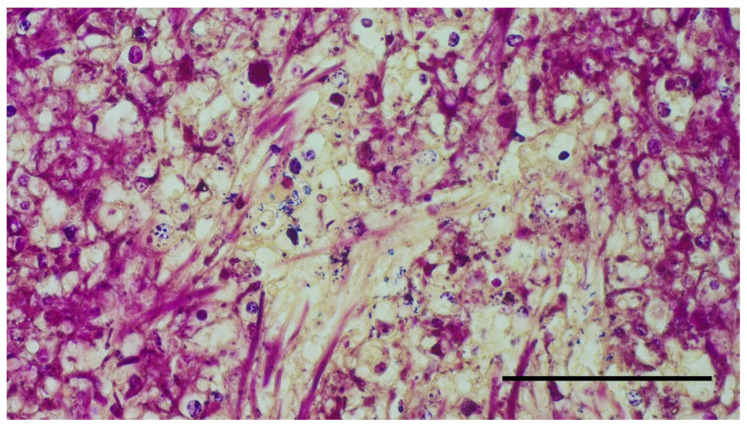
Aggregates of intralesional rod-shaped Gram+ bacteria in the liver. Magnification 400×, scale bar: 1 mm.

Samples of lung, heart, liver, pancreas, spleen, kidney, and urinary bladder were collected, immediately stored in 10% buffered formalin, and then processed for routine histopathology. Formalin-fixed paraffin-embedded (FFPE) tissues were sectioned using HistoCore Autocut microtome (Leica Microsystems, Wetzlar, Germany) with a thickness of 3 µm. The tissue sections obtained were routinely stained with hematoxylin-eosin (HE) and with Gram stain kit (Sigma Aldrich-77730, St. Louis, MO, USA) following standard procedures. Additional sections of spleen and liver were carried out and stained with the Ziehl–Neelsen (ZN) technique (Bio-Optica Milano Spa—05-M20007, 05-M23001, Milano, Italy) for the identification of acid-fast bacteria. Histopathological examination revealed variably sized, nodular, multifocal to coalescing lesions consistent with granulomas in lungs, liver, pericardium, myocardium, pancreas, spleen, kidneys, and within the wall of the urinary bladder associated to necrotizing cystitis. Lesions were characterized by a central aggregate of eosinophilic amorphous material mixed with extravasated erythrocytes, cellular debris and karyorrhectic heterophils (necrosis) surrounded by numerous epithelioid macrophages and multinucleated giant cells, followed by a rim of reactive fibroblasts and collagen bundles infiltrated by a moderate number of lymphocytes and plasma cells (heterophilic granuloma) (Figure 5A,B). Gram stain showed aggregates of cocci and rod-shaped Gram+ bacteria within the lesions in all tissues tested (Figure 6). The ZN stains were negative for acid-fast organisms.

Given the similar aspect of the nodular lesions on all organs, bacteriological analyses were performed immediately after collection for only the liver and heart. Samples were streaked on 5% blood agar and Mac Conkey and Thiosulfate Citrate Bile Sucrose (TCBS) agar plates; the first were incubated for 24–48 h at 25 °C, and the others for 24 h at 37 °C. Colony growth was detected from the blood agar plates; specifically, colonies from the liver had a homogeneous morphology, while colonies from the heart showed two different morphological types, suggesting in the latter case the isolation of two different microorganisms. *L. monocytogenes* was isolated, and matrix-assisted laser desorption/ionization time-of-flight (MALDI—TOF Biotyper; Bruker Daltonics Inc., Billerica, MA, USA) mass spectrometry was used for the identification of isolated species, revealing the presence of liver (Strain S1) and heart (Strain S2), and *Enterococcus faecium* in the heart. Thus, *L. monocytogenes* isolates were serotyped according to the method described in the Bacteriological Analytical Manual [14] using commercial anti O and H antisera (Denka Seiken, Tokyo, Japan). The result showed that both strains belonged to the same serotype (4b), phylogenetically associated with lineage I.

Genomic DNA was extracted from *L. monocytogenes* strains S1 and S2 using the Maxwell HT 96 gDNA Blood Isolation System (Promega, Madison, WI, USA) following the manufacturer’s instructions, and libraries were prepared using the Illumina^®^ DNA Prep (M) Tagmentation kit (catalogue number 20060059, Illumina, San Diego, CA, USA) following the manufacturer’s protocol and sequenced on NextSeq 500/550 platform (Illumina) producing 150 × 2 bp paired-end reads. Reads were trimmed with Trimmomatic ver. 0.38, checked for quality with FastQC v.0.11.5 (https://github.com/s-andrews/FastQC, accessed on 1 February 2018) and assembled using Unicycler ver. 0.4.8 [15] with criteria according to the ECDC analysis pipeline [16]. In silico *Multi Locus Sequence Typing* (MLST) with the Pasteur scheme and presence/absence of genes conferring virulence, resistance to disinfectants/metal and antibiotics were determined using the Pasteur BIGSdb for *L. monocytogenes* (https://bigsdb.pasteur.fr/listeria/, accessed on 8 July 2022) with the Pasteur scheme.

The genomes of both *L. monocytogenes* strains S1 and S2 belonged to the same serogroup, namely IVb, Sequence Type 6 (ST6) and Clonal Complex 6 (CC6). Five antibiotic resistance genes were detected in both S1 and S2 genomes: *fosX*, *lin*, *mprF*, *norB*, and *sul* (conferring resistance to fosfomycin, lincomycin, oxacillin, cephalosporins, and sulfamethoxazole). Conversely, no genes of resistance to disinfectants or metals were found.

**Figure 7:**
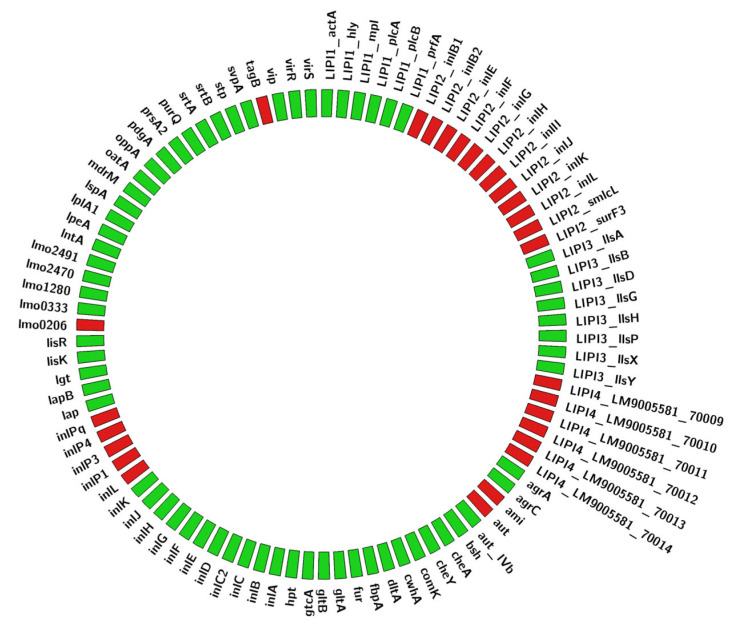
Graphical representation of the virulence factors of the isolated *L. monocytogenes*. The present virulence genes are reported in green, while the missing ones are reported in red.

The presence of 66 out of 93 virulence genes listed in the Pasteur BIGSdb for *L. monocytogenes* implicated in pathogenetic mechanisms (e.g., invasion, adherence) was detected in both S1 and S2 genomes (see details in Figure 7), outlining a virulence profile common to ST6 strains [17]. More precisely, two Listeria pathogenicity islands (LIPI) were found: LIPI-1, which includes genes (*prfA*, *plcA*, *hly*, *mpl*, *actA*, *plcB*, *orfX*) essential for survival, intracellular growth, and spread from cell to cell [18], and LIPI-3, which encodes for listeriolysin S (LLS) that alters intestinal microbiota composition [19]. On the contrary, genes belonging to the LIPI-4 (from LM9005581_70009 to LM9005581_70014 genes in Figure 7), associated with hypervirulence [20], and to the LIPI-2, associated to *L. ivanovii*, were absent.

## 3. Discussion

Here we reported the clinical case of a loggerhead sea turtle with internal lesions similar to those of the systemic granulomatous disease caused by *L. monocytogenes* in an adult *Caretta caretta* found along the Adriatic coast in summer 2021 in Italy, described by Di Renzo and colleagues [11].

The animal was administered a single dose of enrofloxacin, a bactericidal fluoroquinolone antibiotic, with effects for both Gram positive and negative bacteria, also active against *Mycobacterium* spp. [21], comprising *M. chelonae*. Thus, we cannot exclude that this might have influenced the growth of other bacteria. In addition, it is typical for reptiles to respond with granulomatous reaction to infectious agents, particularly to *Mycobacterium* spp. infection [22]. However, considering both the detection of intralesional Gram positive bacteria and the negative histochemical result of the ZN stain, we excluded the pathological involvement of acid-fast bacteria. Indeed, ZN staining would have allowed identification of dyed microorganisms within stained tissue sections. On the other side, the laboratory analyses revealed the presence of *E. faecium* in very low amounts within cultures from the heart, while *L. monocytogenes* was isolated in high quantities from both liver and heart.

Based on previous evidence, we have concentrated our attention only on *L. monocytogenes* as the etiological agent, but we cannot conclude that it was the causative agent of the death of the animal, nor is it possible to determine if the weakness of the animal was caused by *L. monocytogenes* or vice versa. Furthermore, the animal was surely suffering a condition of disseminated listeriosis. Indeed a general condition of granulomatous lesions on all internal organs was evident, as witnessed by Di Renzo [11]. The weight was low, and this was in line with the symptoms described in the few cases of reptilian listeriosis in which affected individuals displayed nonspecific signs of disease, primarily anorexia and obtundation [10]. Regarding the latter, the animal when rescued was trying to reach the inland outside of the season for laying eggs. The gastrointestinal helminths in the lumen of the esophagus and stomach were retrieved, but could not be further characterized due to technical reasons. Despite this, the infection is described throughout all size classes with a prevalence for this age class of 50% [23]. Although cases of listeriosis in reptiles are rare, some cases of septicemia associated with granulomatous systemic response concurrent with *L. monocytogenes* infection are reported [24,25,26].

Regarding the finding of *Enterococcus faecium*, enterococci are in general commensal bacteria of the oral cavity, genitourinary, and gastrointestinal tracts of humans and other animal species comprising reptiles [27], leading to the assumption that a cross contamination during sampling might have occurred. Another reason is that the microorganism was not recovered from both internal organs, since only the liver tested positive, but this might be related either to the contamination or to the antimicrobial administration. However, it should be mentioned that, in humans, enterococci are among the leading causative microorganisms of bloodstream infections, particularly in healthcare settings and in elderly, fragile, and immunosuppressed patients [28].

*Listeria monocytogenes* is an important microorganism from a One Health perspective, with unique potential to spread from cell to cell, thereby crossing blood-brain, intestinal, and placental barriers, causing fatal infections in humans and in a wide range of animals. Since its first identification in 1985, the presence and implications of *L. monocytogenes* in food and food processing environments have been extensively studied, but its ecology in natural habitats remains poorly understood [29]. The peculiar *L. monocytogenes* transmission cycle between humans, animals, and environment through sewage and food-processing plants likely increases the load of *L. monocytogenes* in different habitats [30]. Particularly, the marine environment is subjected to a variety of contaminants: in addition to native marine and estuarine microorganisms, other species can be introduced to the seas as the result of human activity [31]. This is particularly true related to our findings, since we identified a *L. monocytogenes* isolate of ST6 from a deceased loggerhead sea turtle. This sequence type is increasingly associated with outbreaks related to food in Europe [20,32,33] and around the world, as is the case of the largest listeriosis outbreak that took place in South Africa, where it affected 937 people with a 27% mortality rate [34,35]. Our results report antimicrobial resistance genes that are known to be present in almost all *L. monocytogenes* ST genomes [36,37]. The presence of an ST6 isolate carrying virulence genes involved in invasion and adherence mechanisms could be relevant in wildlife animals. In fact, they could play an active role as carriers of possibly pathogenetic and virulent strains, and contribute to the distribution of *L. monocytogenes* in the environment [38,39]. This is even more true when considering species such as *Caretta caretta*, which can migrate for hundreds or thousands of kilometers [6,40].

None of the investigations performed to date by means of metagenomics demonstrated the presence of *L. monocytogenes* in either the feces and intestinal mucosa of loggerhead sea turtles, [41,42,43,44] nor have they indicated that the microorganism may be among the zoonotic pathogens eventually transmitted by these animals [45]. On the other side, numerous descriptions of *L. monocytogenes* in marine molluscs and fish are present in the Literature [7,46,47]; specifically, among these animals, the ST6 has been reported [29]. Among animals, this sequence type has additionally been described in an aborted water buffalo fetus in Southern Italy [48] and from the liver of a white-faced saki (*Pithecia pithecia*) that died following septicemic listeriosis [49].

Humans are increasingly invading wildlife habitats, enhancing the frequency of human–wildlife contacts and associated pathogen transfer events. To date, some paths are neither considered, nor completely understood, thus our findings emphasize the value of zoonotic pathogen surveillance among wildlife that share some geographical domain with human habitats and the food supply chain to support One Health. Surveillance of wildlife disease may provide information on domestic and wild animal morbidity and mortality, and allow to identify changes in patterns of disease occurrence over time. Elusive aquatic species that present criticalities such as *Caretta caretta* might be tested by means of environmental (e) DNA [50], which allows to perform sea turtle population genetic studies as well as pathogen monitoring. This might also be applied for evaluating the potential sentinel of this species for zoonotic and terrestrial pathogens in the marine environment [48], as suggested for other aquatic species, as an indicator of the anthropic pressure.

## 4. Conclusions

Our previous reported findings suggest that the marine environment might contribute to *L. monocytogenes* circulation and should also be considered a possible source. Taken all together, clinicians, pathologists, and microbiologists should also consider the occurrence of *L. monocytogenes* infections in *Caretta caretta*, and pose it in differential diagnosis mainly with infection by *Mycobacterium chelonae*, but also evaluate the possibility that further manifestations might be possible.

In addition, the concentrated evidence of three cases in a small time-period may raise an issue regarding a possible modification of the *equilibrium* among the environment and the pathogen, posing threats for a possible zoonotic transmission through contact with the stranded animals or a higher risk of contamination of fish and fishery products.

## Data Availability

Genomic data for this study have been deposited in the European Nucleotide Archive (ENA) at EMBL-EBI under accession number PRJEB60797 (https://www.ebi.ac.uk/ena/browser/view/ PRJEB60797, accessed on 1 April 2023).
